# Experimental and Numerical Study of Laser Beam Welding of PBT-G30 for Electronic Housings in Automotive Applications

**DOI:** 10.3390/polym17192662

**Published:** 2025-10-01

**Authors:** Luiz R. R. Silva, Paulo D. P. Nunes, Eduardo A. S. Marques, Ricardo J. C. Carbas, Lucas F. M. da Silva

**Affiliations:** 1 Departamento de Engenharia Mecânica, Faculdade de Engenharia (FEUP), Universidade do Porto, Rua Dr. Roberto Frias, 4200-465 Porto, Portugal; luiz.silva@ifes.edu.br (L.R.R.S.); lucas@fe.up.pt (L.F.M.d.S.); 2 Ciência e Tecnologia do Espírito Santo (Ifes), Instituto Federal de Educação, Rodovia BR Norte Km 58, 101-Litorâneo, São Mateus 29932-540, ES, Brazil; 3 Instituto de Ciência e Inovação em Engenharia Mecânica e Engenharia Industrial (INEGI), Campus da FEUP, R. Dr. Roberto Frias 400, 4200-465 Porto, Portugal; pnunes@inegi.up.pt (P.D.P.N.);

**Keywords:** laser welding, PBT GF30, housing, automotive, numerical modeling

## Abstract

This study investigates the application of laser spot welding to join protective housing components in the automotive electronics industry. The PBT GF 30 components were joined using two primary configurations: a purely overlapping joint and a top-overlap joint, both autogenous (i.e., without filler material). To complement the experimental analysis, a numerical model, previously validated for a simpler joint configuration, was adapted and applied to configurations beyond the overlapping and top-overlap joint, more representative of practical automotive industry components. The results demonstrated that butt-overlap joints exhibited significantly higher strength (85% increase) than purely overlapping joints. This enhancement is attributed to the combined effect of normal and shear stresses in the top-overlap configuration, whereas purely overlapping joints rely solely on shear stress. The validated numerical model accurately predicted the experimental results, including displacement and force values. While minor deviations were observed, the numerical model’s predictions converged within the average experimental values and standard deviation, demonstrating that such a model can be used to precisely design laser-welded joints for similar applications.

## 1. Introduction

With the increasingly tighter regulations and demands facing the automotive industry, one of the key priorities of the companies operating in this sector has been the usage of lighter and more resistant materials, in an effort to reduce weight and energy consumption, while ensuring maximum functionality [1]. While most of the effort is being devoted to new structural designs, other components, such as electronic systems, have also been adopting lighter materials. However, according to Begum et al. [2] and Patil et al. [3], for these smaller components, especially electrical component protection, it is also necessary to analyse a wide range of parameters, such as flame protection, flame retardancy, and water tightness, ensuring that the systems can operate without issues. Due to their versatility, polymers dominate this field of application, especially those that employ short-fiber reinforcement, increasing their performance at a relatively low cost and without adding complexity to the manufacturing process [4,5,6].

Among all the possible polymeric matrices available for these short-fiber composites, polybutylene terephthalate (PBT) has multiple advantages compared to other polymers, such as its good chemical resistance and electrical properties [7], is relatively hard, and shows limited water absorption [8]. It also has very good resistance to dynamic stress [9] and excellent thermal and dimensional stability [10]. PBT is relatively economical and simple to manufacture due to fast crystallization and fast cooling. Accordingly, it finds extensive usage in electronics protection housings, but it can also be used in many other applications with complex geometries [11], such as fog lamp housings and bezels, sun-roof front parts, locking system housings, door handles, bumpers, carburetor components, among many others. Characterization of such materials is often performed using non-contact methods, such as Digital Image Correlation (DIC). DIC is a non-contact optical technique that measures full-field surface displacements and strains by precisely tracking a unique, random speckle pattern applied to a material’s surface as it is being loaded. The technique is used in two main forms, 2D DIC (for in-plane, flat-surface analysis) and 3D DIC (for complex, three-dimensional deformation analysis using a stereo camera setup), and is widely valued in industry for its accuracy and versatility in evaluating material behavior, validating engineering simulations, and improving product reliability.

The application of reinforced PBT, mainly with glass fibers (PBT GF-30, reinforced with 30% glass fiber by weight), has increased in the market due to the excellent mechanical properties it provides. It is also very important in dynamic applications, providing high hardness and a low friction coefficient, reducing wear [12]. Furthermore, it is relatively easy to recycle, placing itself as a more sustainable polymer according to Pegoretti et al. [13].

When using such materials, it is also important to master a set of joining processes that allow us to assemble them into larger components. Of course, for thermoplastic polymers, laser welding is almost always one of the most significant joining methods, also supported with ultrasonic welding and adhesive bonding (Silva et al., 2021) [14]. Furthermore, compared to other joining methods like ultrasonic or hot-plate welding, laser welding is a non-contact, precise process that produces clean and aesthetically pleasing joints, making it suitable for sensitive applications despite its higher initial cost and specific material requirements. Looking specifically at the laser welding application, the authors further developed and optimized a model for predicting the performance of laser-welded joints in PBT GF30, demonstrating its suitability for simple joint geometries. Laser welding of polybutylene terephthalate (PBT) often utilizes through-transmission laser welding (TTLW), a method where a laser beam passes through a transparent PBT part and is absorbed by an opaque one at the joint interface [15]. A key challenge is PBT’s semi-crystalline structure, which scatters near-infrared laser light and reduces energy transmission; researchers overcome this by either developing new laser-optimized PBT grades with refined crystal structures or, more commonly, by incorporating laser-absorbing additives like carbon black (CB) into the opaque part [16]. The quality of the weld is highly dependent on controlling process parameters such as laser power, scanning speed, and clamping pressure, which are crucial for ensuring proper melting, heat transfer, and molecular diffusion at the joint [16]. The work of Okuizumi et al. [17] introduces the use of absorption energy as a parameter in thermal diffusion suitable for predicting experimental results in laser welding of PBT, stating that maximum welding strength values correlated well with absorption energy values and concluding that the absorption energy in thermal diffusion is an essential parameter for predicting experimental results in laser welding. Modeling the mechanical performance of laser-welded polymers is a challenging process that typically involves a multi-scale, coupled thermo-mechanical finite element approach: thermal models first predict transient temperature fields—key for estimating the melt depth, heat-affected zone [18], and potential thermal degradation [19]—followed by mechanical simulations that incorporate temperature-dependent material behavior (elastic, plastic, and viscoelastic properties) to evaluate residual stresses, distortions, and stress concentrations at the joint. These simulations often employ sequential coupling, using thermal outputs as input for mechanical analyses and exploiting constitutive models such as elastoplasticity with isotropic hardening or viscoplastic formulations to capture polymer response during cooling. The work of Fernandes et al. [20] demonstrated mechanical finite element modeling of tensile tests on welded thermoplastics, using a unified mesh, elastoplastic constitutive behavior, and self-contact to identify stress concentrations and potential failure zones. Nonetheless, although PBT-GF30 components are already industrially manufactured using laser welding, there is a significant research gap in understanding how to more effectively laser weld and, more significantly, model the performance of real-world PBT-GF30 components, especially given the complexity of joints found in industrial applications and the specifications of welding a short-fiber composite. While existing studies often use simplified specimen configurations, this work aims to bridge that gap by developing a new methodology to predict the performance of non-standard laser-welded joints through an experimental procedure and a validated model directly applied to components with geometries which deviate from standard joint configurations, such as butt-joints or single-lap joints.

## 2. Experimental Methodology

**Figure 1:**
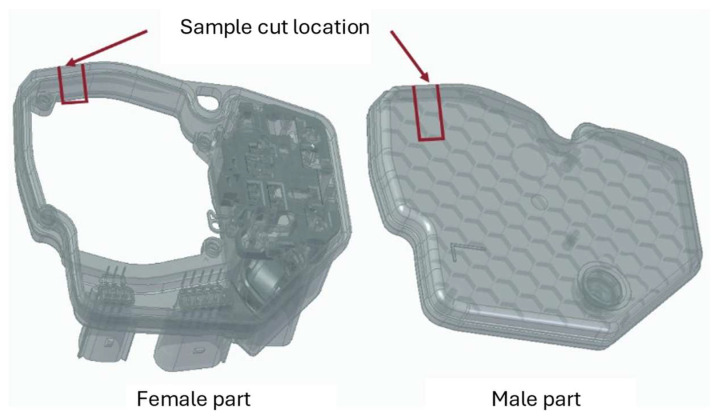
Illustration of the electronics housing, showing the location of the cut made to extract the test specimens.

This work, based on industrial demand from the automotive sector, seeks to join housing components with electronic components. These housings, as shown in Figure 1, are usually composed of two separate halves, which are bonded together to seal the electronic boards placed inside them. However, adhesive bonding has some limitations in what concerns long long-term durability, as water ingress can in some cases damage the bonding interfaces. In this work, laser welding is instead used to achieve this joint, with the aim of providing a more homogenous connection, less susceptible to environmental contamination. To prepare specimens for testing, small substrates were cut from the complete samples of the electronic housing. Since the goal is to assess the performance of the joint and not of the complete housing, 10 mm wide samples were extracted from different areas of the box, allowing them to better study how the parts can be connected, while providing a level of load that was still high enough to allow for testing in conventional electromechanical equipment.

**Figure 2:**
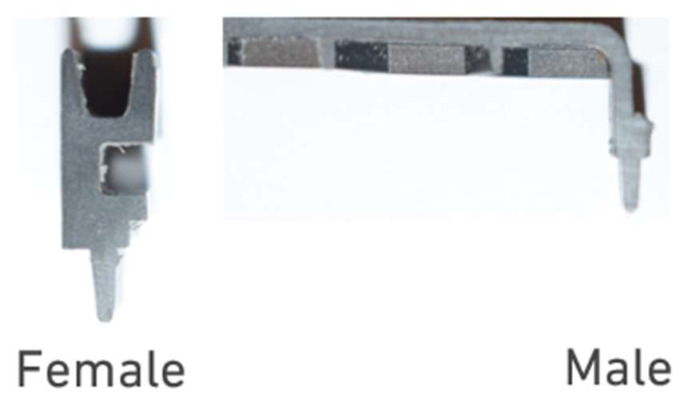
Representation of the cross-sectional view of the joint samples, highlighting the female and male parts.

A cross-section of these cut specimens can be found in Figure 2. It is possible to see that the housing uses a male–female fitting to achieve a precise alignment, which is then usually bonded and sealed with the use of a flexible adhesive.

### 2.1. Laser Welding Process

The equipment used to create the welded joints was a SISMA LM-D 180 (SISMA, Piovene Rocchette, Italy), which uses a Nd:YAG 1064 nm laser source, with a maximum laser power of 80 W. The parameters used to join GF30 PBT overlap joints, in 1 mm thick plates, fillet-welded at their ends, are presented in Table 1. These parameters are based on those used in manual production of such components and offer a relatively slow welding rate, suitable for manual operation. As the material used and the sheet thickness are the same, these procedures and parameters were directly adopted for the current work.

**Figure 3:**
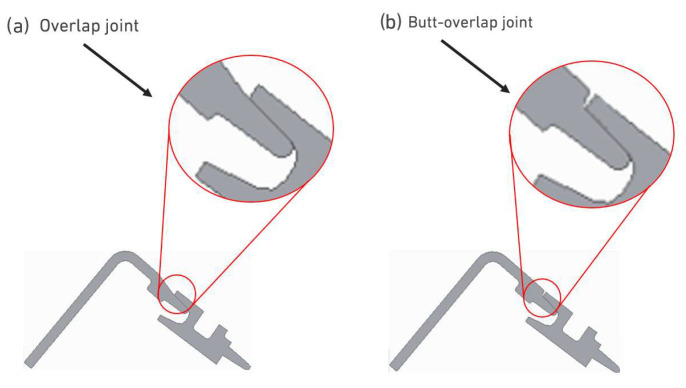
Joint configurations for laser beam welding. (**a**) Overlap joint. (**b**) Butt-overlap joint.

As the experimental testing seeks to provide data that can enable further optimization of the joints found in the full-scale component, in addition to carrying out the test in which the real joint is purely overlapped, a test was also carried out in which the joint is partially overlapped and partially butt-jointed, as shown in Figure 3.

#### 2.1.1. Welding Fixtures

**Figure 4:**
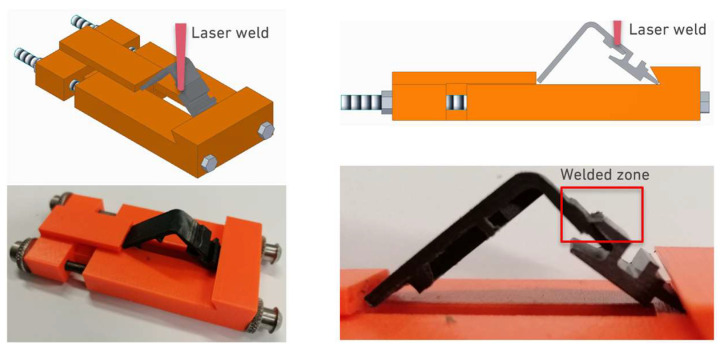
Jig for fixing parts for joining by spot laser beam welding.

In order to join these component-level joints, using non-standard specimens which are difficult to clamp, a specific jig had to be developed to support the welding process, as shown in Figure 4.

This jig, custom-designed and manufactured using additive manufacturing, has the role of ensuring that the substrates are consistently located during welding. This is achieved by tightening two parallel screws, which keep the joint parts aligned and with a positive contact force during the welding process.

#### 2.1.2. Test Fixtures

**Figure 5:**
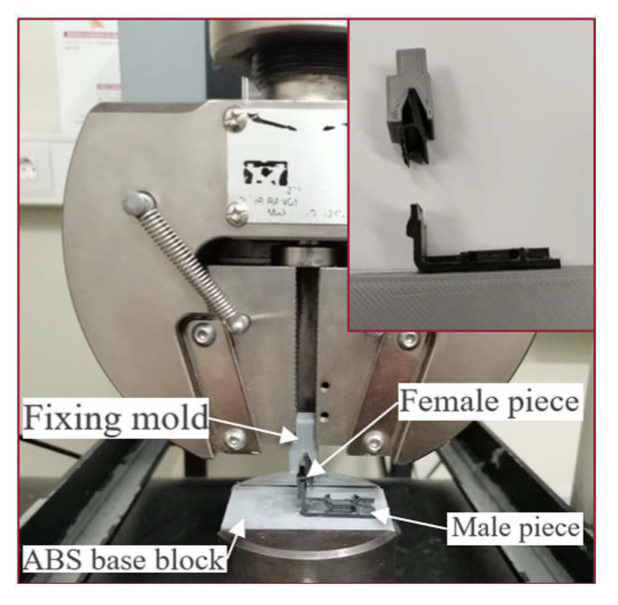
Real joint test fixing configuration.

To allow the specimens to be tested in a universal testing machine, the male part of the joint was fixed to a rectangular ABS block using a structural adhesive, while an additively manufactured fixing mold was bonded to the female part, also using a structural adhesive. This allowed for both ends of the specimen to be clamped to the testing machine, in such a way that the surface of the block was perpendicular to the fixing mold, as shown in Figure 5.

#### 2.1.3. Displacement Measurement

The INSTRON^®^ (Norwood, MA, USA) No 3367 universal testing machine was used to carry out the tensile tests. The crosshead speed setting was 1 mm/min and three tests were carried out for both configurations. For measuring displacement, the digital image correlation (DIC) method was used, based on the studies carried out by Borges et al. (2020) [21]. A single Nikon D5300 camera (Nikon, Tokyo, Japan) was used, equipped with a Nikkor 18–55 mm lens, recording video frames at a resolution of 1920 × 1080 pixels, at a rate of 60 fps. The camera was mounted on a tripod, facing the front of the specimen shown in Figure 5. The sample was speckled using black and white paint and filmed during the test. After completion of the test, the displacement was extracted using the GOE Correlate Professional 2020 software.

## 3. Experimental Results

**Figure 6:**
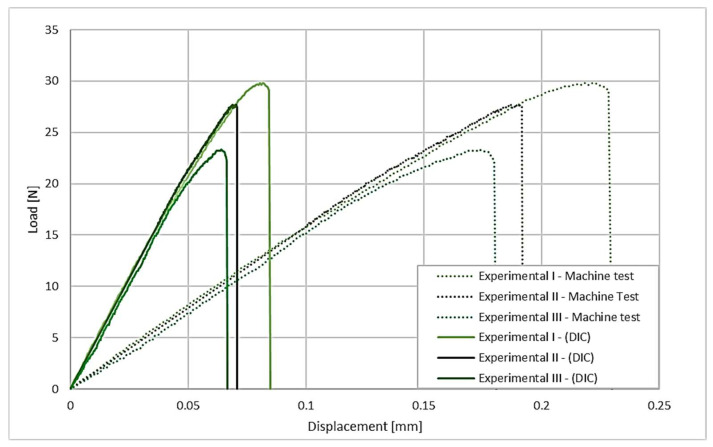
Load displacement curves for the overlapped configuration, showing a comparison between the crosshead displacement and the displacement obtained via DIC.

Experimental testing allows for an accurate comparison of the performance of the two configurations under analysis. Figure 6 shows the results of the overlapped-only configuration, showing the crosshead displacement and the same value corrected using the DIC method.

**Figure 7:**
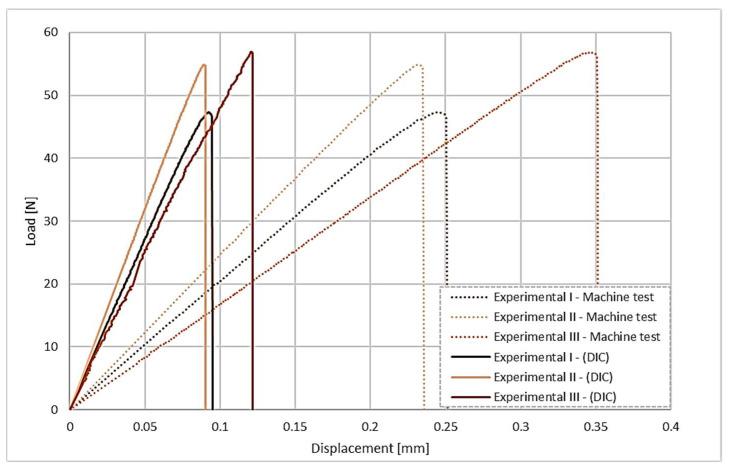
Load displacement curves for the butt-overlap configuration, showing a comparison between the crosshead displacement and the displacement obtained via DIC.

These results show the necessity of carrying out a DIC analysis, since the displacement is extremely small in relation to the size of the joint. Furthermore, the DIC displacement reading is determined solely in the region close to the weld, providing a value that is more representative of the actual joint performance. In contrast, using the testing machine data adds up all the deformation between the joint and the fixtures, as well as the displacement corresponding to flexibility and clearances of the elements in the clamping jaw. For the butt-overlap joints, the results show a similar variation between the displacement measured for the crosshead of the machine and by the DIC method, as shown in Figure 7.

**Figure 8:**
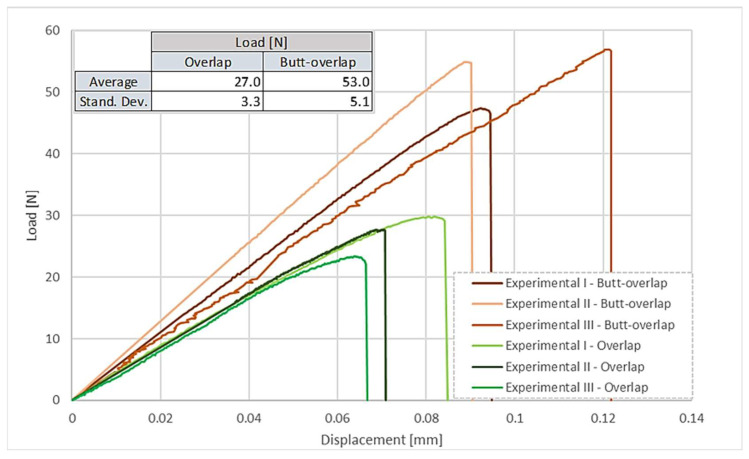
Load–displacement curves of the purely overlapped configuration and the butt-overlap configuration (displacement measured by the DIC method).

When comparing the load displacement curves of the two configurations, as shown in Figure 8, it becomes evident that both the strength and the displacement of the butt-overlap joint configuration are significantly higher than that found for the overlap joint, reaching almost double the peak strength.

This difference is explained in the work of Kagan et al. [22], which describes that, in lap welding, a fused zone is formed both in the overlapped part, which mainly resists shear stress, and in the top region, which mainly resists normal stress. This will increase the overall strength of the joint, especially considering the contribution provided by the fused material that is parallel to the load, which, acting in shear, will make use of the full available area. In contrast, the area where normal stresses are dominant is more susceptible to cracks, defects, voids, and undercuts. Given the results displayed in Figure 8, it becomes evident that, if possible, laser welding in this type of connection should adopt a butt-overlap combination, ensuring maximum performance.

## 4. Numerical Details

**Figure 9:**
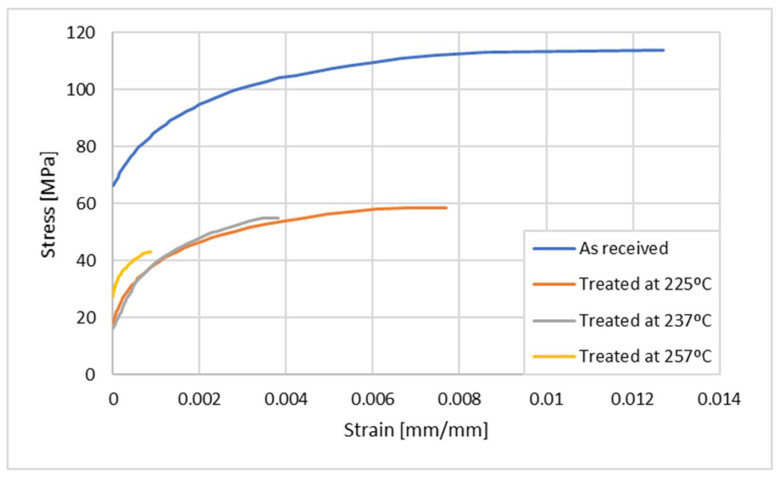
Experimental curves representing the plastic region of PBT GF 30.

As the displacement of the joint has been calibrated using DIC, it becomes feasible to develop a numerical model that is able to precisely model the behavior of the real joint. This can be achieved using the physical and elastic properties of PBT GF30 obtained after exposure to different temperatures, which were experimentally determined and shown in Table 2. Furthermore, the curves shown in Figure 9 provide the deformation and stress in the plastic zone of PBT GF30 and were also used to model the elastoplastic behaviour of this material, detailing the behaviour of PBT GF 30 in three different states: “As received”, “Treated at 225 °C”, “Treated at 237 °C”, and “Treated at 257 °C”.

As shown in Figure 9, there is a significant effect of the exposure temperature (thermal ageing) on the mechanical performance of the material. This is mostly due to two different factors, one stemming from the fact that thermal degradation will damage the polymeric chains, with the second being related to the loss of cohesion around the reinforcing fibers, as the material, in its partial or fully melted state, will flow and create increasingly larger sections where there is limited contact with the fibers, as was also reported in the work of Xu et al. [23]. This degradation is quite severe, as the material is exposed to temperatures significantly beyond its melting point, initiating a degradation process, as reported by Xu et al. This degradation can take the form of breakage of polymer chains, oxidation, and even the presence of voids and gaps in the bulk of the material. This greatly reduces the load transmission to the fibers and thus reduces the strength of this short-fiber composite. Stiffness is also reduced, as the contribution of the fibers to the stiffness of the composite is lowered.

The approach proposed for modelling follows a ductile damage model [24], which has been extensively used by the authors for other welding and joining-related applications [25,26]. Table 3 shows the failure deformations, triaxialities, deformation rates, and displacements up to failure for the implementation of ductile damage. Finally, Figure 9 illustrates the stress and strain curves in the plastic regions of the materials, detailing the characteristics of PBT GF 30 in three different states: “As received”, “Treated at 225 °C”, “Treated at 237 °C”, and “Treated at 257 °C”.

Following the refinement of the characterization of the gradient of the PBT GF30 weld carried out through characterization by Raman spectroscopy, it is possible to establish the percentage of the welded zone that follows a given material behaviour (stemming from exposure to a given temperature range), resulting in the gradient of mechanical properties at the three temperatures under study, shown in Table 4.

### 4.1. Microscopical Analysis

**Figure 10:**
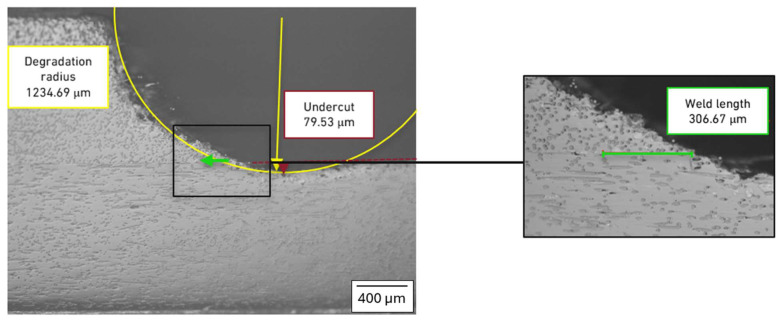
Geometrical characterization of the weld in the purely overlapping configuration. Yellow lines correspond to the degradation radius, red line to the undercut dimensions and green lines to the weld length.

**Figure 11:**
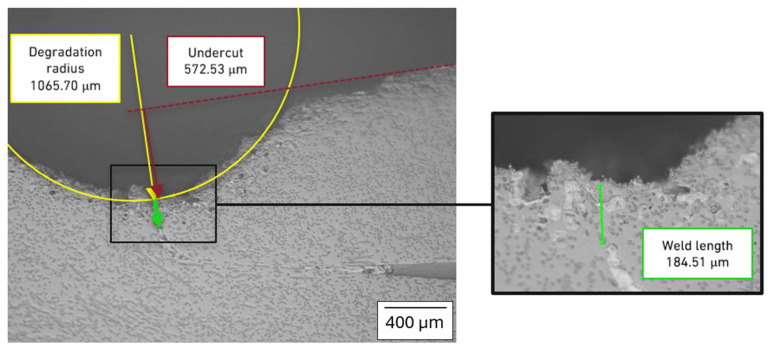
Geometrical characterization of the weld in the butt-overlap configuration. Yellow lines correspond to the degradation radius, red line to the undercut dimensions and green lines to the weld length.

In order to characterize the cross-section geometry and to measure the effective length of the weld, a micrographic analysis was carried out for the lap joint (Figure 10) and the butt-overlap joint (Figure 11).

This exercise also shows that while the effective weld length for the purely overlapped joint is higher, the undercut is also significantly higher, which justifies the low performance of this joint. In this case, the undercut is creating a reduction in the area available for load transfer, thus facilitating the generation of higher stresses and the eventual failure of the joint. The degradation radius stemming from exposure to the laser beam is quite similar, which is also expected, given that the same welding parameters were used for both cases.

### 4.2. Modelling Details

**Figure 12:**
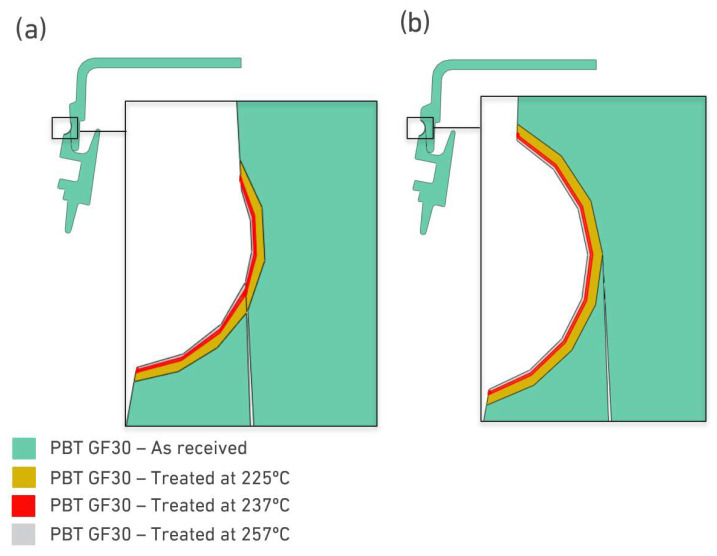
Representation of distribution of different material properties along the joint. (**a**) Overlap joint and (**b**) Butt-overlap joint.

The models of the joint were generated in Abaqus, using the geometric measurements presented in Figure 10 and Figure 11, using the percentual distributions of the mechanical properties shown in Table 4. The model for the purely overlapped configuration is shown in Figure 12a, while Figure 12b shows the butt-overlapped configuration, highlighting the different properties applied to different regions around the welded area.

**Figure 13:**
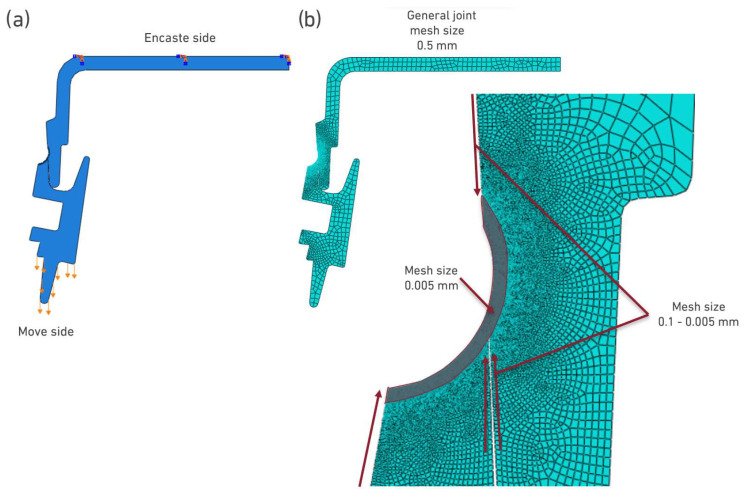
Schematic representation of the joint model. (**a**) Boundary conditions, with orange arrows representing the boundary conditions and (**b**) Mesh size, red lines indicate the mesh dimensions used.

Once the properties were defined, the boundary conditions for the simulation of the joints were configured. In both configurations, the upper zone (male part) was fully fixed with an encastre, with an imposed movement to the female part, as shown in Figure 13a. In what concerns the element size, the joint generally meshed with 0.5 mm elements and refined in the welding zone with 0.005 mm elements, both in the layers with the different properties (corresponding to different exposures of the material to temperature) and in the regions adjacent to these layers, shown in Figure 13b.

## 5. Numerical Results

**Figure 14:**
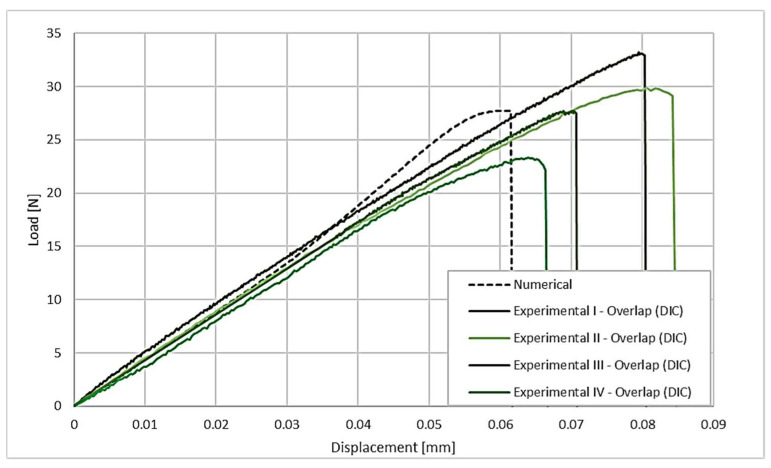
Comparison between experimental load–displacement curves (corrected by DIC) and numerical curves for the purely overlap configuration.

To validate the model, the simulation was run for both configurations. The numerical results for the model for the purely overlapped configuration are shown in Figure 14, where the dotted line represents the numerical model results.

**Figure 15:**
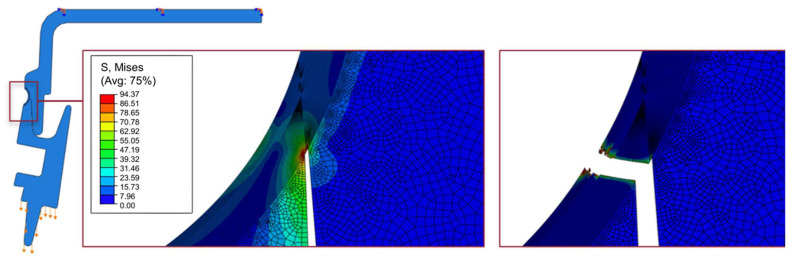
Numerical characterization of the von Mises stress field before failure and representation of the failure mode for the purely overlapped configuration. Arrow represent the loading direction and boundary conditions.

The numerical model for the overlapped joint has good agreement with the experimental data for the maximum strength. Displacement at failure, mainly due to some additional flexibility in the fixation blocks used in the experimental setup, although mitigated by the usage of DIC, still amounts to a non-negligible percentage. Analyzing the fracture process (Figure 15) for the joint in the purely overlapped configuration, it is noticeable that there is an asymmetry in the stress field, showing a concentration of stress on the right side at the bottom, stemming from the greater shear stress generated in that region of the joint. This is caused by the nature of the joint geometry and would necessitate the redesign of the connection, with a more symmetrical connection between the upper and the lower components.

**Figure 16:**
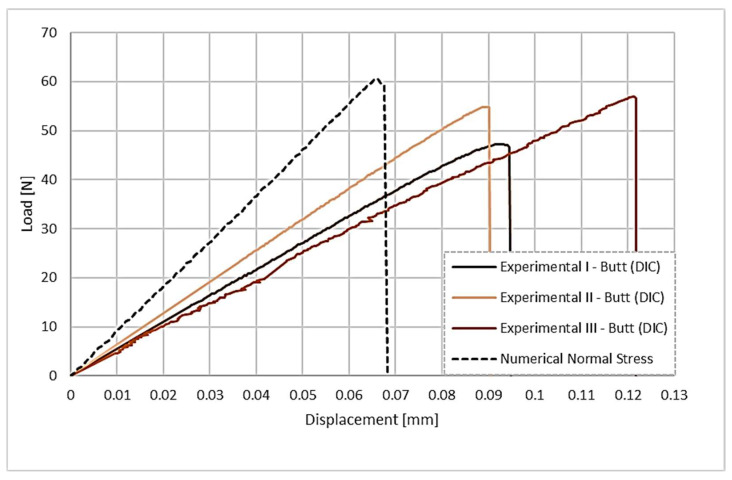
Comparison between experimental load–displacement curves (corrected by DIC) and numerical curves for the butt-overlap configuration.

Comparing the numerical simulation for the butt-overlap joint with the experimental results shown in Figure 16, the failure load has a good level of agreement. However, the displacement expressed in the simulation is again slightly lower than the displacement of the experimental curves.

**Figure 17:**
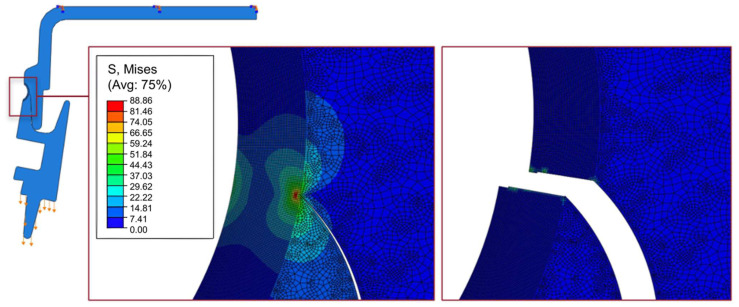
Numerical characterization of the von Mises stress field before failure and representation of the failure mode for the butt-overlap configuration. Arrows represent loading direction and boundary conditions.

The damage in the model for the butt-overlap joint is driven by stresses acting mostly in the normal direction, exhibiting a typical crack opening process. This is due to the fact that there is a more symmetrical stress field at the crack tip, as shown in Figure 17. In fact, in addition to supporting a large amount of load through shear, the same joint is also able to support normal strength, delaying damage and crack formation, explaining the increased performance over the purely overlapped joint.

## 6. Conclusions

Laser welding has proven to be a feasible and effective joining technique for PBT GF30 components, particularly for applications such as automotive electronic housings. This study successfully demonstrated the effectiveness of this method and provided key insights into the mechanics of the resulting joints.

- The butt-overlap joint configuration created a stronger connection than a purely overlapped one, as it could resist both shear and normal stresses simultaneously.
- Digital Image Correlation (DIC) was validated as a robust method for accurately evaluating joint performance by providing precise displacement data.
- Elasto-plastic numerical models with ductile damage formulations were found to be a practical design tool for these joints, showing good convergence in predicting failure loads. However, they faced challenges in accurately matching the real specimen’s overall compliance.

While these models offer a valuable solution for predicting joint performance, future research should focus on refining them to better capture the real-world compliance of the specimens. Additionally, optimizing laser parameters like pulse frequency and advance rate, along with exploring process robotization, could further enhance joint strength and efficiency for industrial applications.

## Figures and Tables

**Table 1:** Optimized parameters and spot laser welding settings of PBT GF30.

Power	64 W	Scheme picture 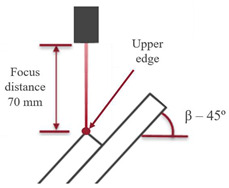
Inclination	1.5 mm	
Aim position	1.5 ms	
Frequency	Manual operation	
Speed rate		
Focus distance	See scheme	
Inclination		
Aim position		

**Table 2:** Main physical and mechanical properties of PBT GF30 in different states.

Material [PBT GF30]	Young’s Modulus [MPa]	Poisson Ratio	Density [g/cm^3^]
As received	8398	0.45	1.31
Treated at 225 °C	5317	0.42	1.31
Treated at 237 °C	5183	0.42	1.31
Treated at 257 °C	4826	0.42	1.31

**Table 3:** Properties of the PBT GF30 for implementing ductile damage.

Material	Fracture Strain [mm/mm]	Stress Triaxiality	Strain Rate [s^−1^]	Displacement at Failure [mm]
As received	0.011492123	−0.33	0	0.001
Treated at 225 °C	0.006559261	−0.33	0	0.001
Treated at 237 °C	0.003570756	−0.33	0	0.0007
Treated at 257 °C	0.000821712	−0.33	0	0.0007

**Table 4:** Representative percentages of the mechanical properties within the weld zone.

External Layer	Middle Layer	Internal Layer
Treated at 225 °C	Treated at 237 °C	Treated at 257 °C
61.60%	15.40%	23%

## Data Availability

The original contributions presented in this study are included in the article. Further inquiries can be directed to the corresponding author.
